# Post–Column Guanosine Addition as a Screening Tool in the Search for Effective G–Quadruplex Binders—A Case Study of *Achyrocline satureioides* Phenolic Compounds

**DOI:** 10.3390/ijms26094312

**Published:** 2025-05-01

**Authors:** Olga Stężycka, Magdalena Frańska, Damian Nowak, Marcin Hoffmann, Małgorzata Kasperkowiak, Monika Beszterda-Buszczak

**Affiliations:** 1 Institute of Chemistry and Technical Electrochemistry, Poznań University of Technology, Berdychowo 4, 60-965 Poznań, Poland; olga.stezycka@doctorate.put.poznan.pl; 2 Faculty of Chemistry, Adam Mickiewicz University, Uniwersytetu Poznańskiego 8, 61-614 Poznań, Poland; damian.nowak@amu.edu.pl (D.N.);; 3 Center for Advanced Technologies, Adam Mickiewicz University, Uniwersytetu Poznańskiego 10, 61-614 Poznań, Poland; 4 Department of Food Biochemistry and Analysis, Poznań University of Life Sciences, Mazowiecka 48, 60-623 Poznań, Poland; monika.beszterda@gmail.com

**Keywords:** guanosine adducts, phenolics, electrospray ionization, mass spectrometry, post-column addition, liquid chromatography

## Abstract

Polyphenols make a numerous and diverse group of plant secondary metabolites exhibiting remarkable anticancer activities, often attributed to their G-quadruplex binding properties. Therefore, there is a need to develop a high–throughput screening assay which would permit the evaluation of polyphenols’ binding properties toward G-quadruplex. As deoxyguanosine and guanosine are essential and key building blocks of G-quadruplexes, the stabilities of their adducts with polyphenols may reflect the stabilities of polyphenols–G-quadruplex adducts. In this study, deoxyguanosine/guanosine post-column addition experiments have been performed during HPLC-MS analysis of *Achyrocline satureioides* extract. The stabilities of the deoxyguanosine/guanosine adducts with 3-*O*-methylquercetin-7-*O*-glucoside, 4′-hydroxydehydrokawain-4′-*O*-glucoside, and 3,5-di-*O*-caffeoylquinic acid—compounds identified in the *Achyrocline satureioides* extract—have been tested by using collision-induced dissociation ‘in-source’. The obtained results show that the identified compounds form more stable adducts with deoxyguanosine and guanosine than the standards used for comparison, namely isoquercitrin and rutin. The performed molecular docking provided some insight into the structure of the adducts and revealed that multiple interactions are of key importance for their stabilities.

## 1. Introduction

Post-column addition-based liquid chromatographic analysis enables a significant increase in sensitivity and selectivity (mainly as a result of a post–column derivatization), and also permits carrying out high-throughput bioaffinity screening assays [1,2,3,4]. For example, the post-column addition of CH_3_COONa or AlCl_3_, as UV-shift reagents during the HPLC-UV analysis of flavonoids, can be employed to determine the positions of hydroxyl groups on the flavone skeleton [5,6,7,8,9], and the post-column addition of 2,2-diphenyl-1-picrylhydrazyl radical (DPPH) can be applied to determine the antioxidant activities of the phenolic compounds [10,11,12]. A similar approach can also be followed for profiling the DNA-binding activities of phenolic compounds [13].

Currently, there is a growing interest in compounds of natural origin in the context of their use as effective G-quadruplex binders, as described in detail in review papers [14,15,16]. Therefore, the effective and efficient screening methods of evaluation of binding properties of natural compounds toward G-quadruplexes are also of interest [17,18,19,20]. Among the natural compounds, polyphenols, which are well-known for their health benefit properties, show remarkable anti–cancer effects which may often be attributed to their G-quadruplex binding properties [21]. Therefore, it is desirable to develop a high–throughput screening assay that would permit evaluation of polyphenols’ binding properties toward G-quadruplex [22,23].

*Achyrocline satureioides* is a herbaceous plant native to South Africa that exhibits very interesting pharmacological properties, including anticancer ones, attributed to its phenolic compounds [24,25]. Among these phenolic compounds are those which are quite common in nature, e.g., 3-*O*-methylquercetin [26,27,28] or dicaffeoylquinic acids [29,30], as well as those which are very rare, e.g., achyrobichalcone [31,32,33] or 4′-hydroxydehydrokawain [34].

**Scheme 1:**
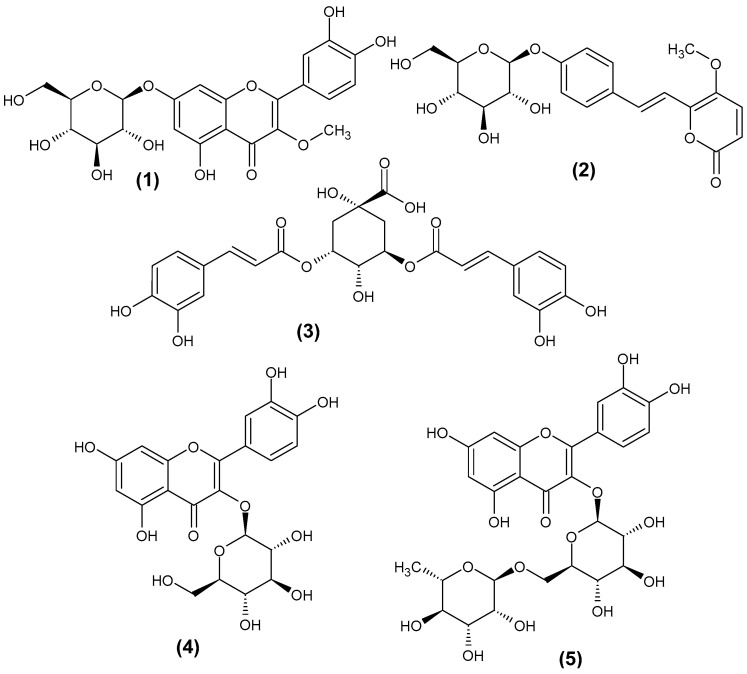
Structures of the studied phenolic compounds.

In this work, we performed the deoxyguanosine/guanosine post-column addition during HPLC-MS analysis of *Achyrocline satureioides* extract and tested the stabilities of the formed phenolic–deoxyguanosine [M+dG+H]^+^ and phenolic guanosine adducts [M+G+H]^+^ (M stands for a phenolic molecule). Since dG and G are essential and key building blocks of G-quadruplexes, the stabilities of such adducts may reflect the stabilities of phenolic-G-quadruplex adducts. Therefore, the described approach may be an effective tool for screening the natural G-quadruplex binders. Three phenolic compounds identified in the extract were selected to test the stabilities of their adducts with dG and G, namely, 3-*O*-methylquercetin-7-*O*-glucoside—**1**, 4′-hydroxydehydrokawain-4′-*O*-glucoside—**2**, and 3,5-di-*O*-caffeoylquinic acid—**3** (achyrobichalcone was not detected in our sample). Compounds **1**–**3** represent three different classes of phenolics and were characterized by similar retention times, so their adducts with dG and G were obtained in similar conditions (regarding the solvent composition). Furthermore, compounds **1**–**3** were present in the obtained extract in relatively high amounts, which allowed for the formation of the adducts of interest (it is clear that for low-abundant compounds, the adducts may not be formed). For comparison, the standards of isoquercitrin (**4**) and rutin (**5**) were also included in the study (Scheme 1). To the best of our knowledge, the post-column addition has not yet been employed to evaluate the stabilities of the adducts formed between phenolic and nucleoside molecules.

## 2. Results and Discussion

### 2.1. HPLC–MS Identification of Compounds ***1***–***3***

Compounds **1**–**3** were detected in both positive and negative ion modes, and the characteristic product ions were observed at a higher cone voltage (Table 1), fully confirming their structure.

Chromatograms and mass spectra are shown in the Supplementary Materials (Figures S1–S3). As expected, for compounds **1** and **2**, at longer retention times, the free aglycones (3-*O*-methylquercetin and 4′-hydroxydehydrokawain) were also detected (Figure S4).

Compounds **1** and **2** have yielded aglycone product ions formed due to the breaking of glycosidic bonds. Compound **1** has also yielded the product ions formed due to the presence of a methyl group. Among them, the ion [M–H–CH_3_]^−•^ is worth noting, since it is formed through the loss of a methyl radical substituted at the C3-OH group (homolytic bond cleavage) from a deprotonated molecule of flavonoid glycoside. Homolytic bond cleavage is characteristic of flavonol 3-*O*-glycosides [35,36]; therefore, the presence of the product ion [M–H–CH_3_]^−•^ may be regarded as diagnostic, indicating the methylation of the C3-OH group.

Compound **2** has no free phenolic groups (the only phenolic group has been glycosylated). Under the conditions used, the sugar hydroxyl groups are not acidic enough to produce ion [M–H]^−^ at *m*/*z* 405 (sugars are definitely weaker acids than phenols, pKa of phenol is 10, whereas pKa of glucose is much higher [37]). Therefore, in the negative ion mode, compound **2** yielded ions [M+HCOO]^−^ and [M+Cl]^−^, at *m*/*z* 451 and 441, respectively (formic acid has been added to the mobile phase, and chlorides are present as contamination). 4′-Hydroxydehydrokawain is very rare in nature. It was identified in the extract of *Achyrocline satureioides* a long time ago [34]. In this work, both 4′-hydroxydehydrokawain and its glycoside (**2**) were identified.

Compound **3** has yielded product ions formed through the breaking of ester bonds [38]. Its chromatographic peak shape indicates that it is an isomer mixture (Supplementary Materials, Figure S3). However, the negative ion mass spectrum at maximum (rt = 5.45 min) indicates that the main isomer is 3,5-di-*O*-caffeoylquinic acid, because of a higher abundance of product ion at *m*/*z* 191 than that at *m*/*z* 173 [39]. The minor isomer eluted at a slightly lower retention time is probably 3,4-di-*O*-caffeoylquinic acid [29].

Besides compounds **1**–**3**, other phenolics were also detected in the analyzed extract, e.g., the aglycones of **1** and **2** (3-*O*-methylquercetin and 4′-hydroxydehydrokawain). However, it is known that the presence of the sugar moiety increases the stability of the phenolic adducts with G-quadruplex/G-tetrad; therefore, the selection of glycosides (**1** and **2**) seems to be justified.

### 2.2. Stabilities of the Adducts of ***1***–***5*** with Deoxyguanosine and Guanosine

**Figure 1:**
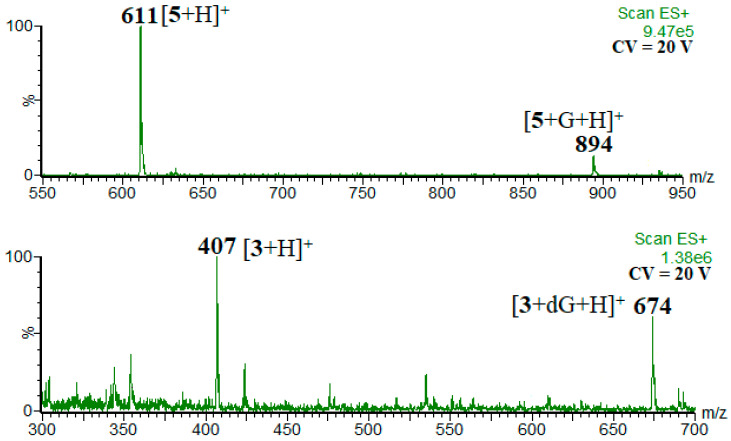
Exemplary mass spectra showing the peaks of the analyzed adducts.

In the post-column addition experiments, the adducts [M+dG/G+H]^+^ were observed (M stands for phenolic molecule). Figure 1 shows exemplary mass spectra.

To compare the adduct stabilities, the plots of the ions ratio [M+dG/G+H]^+^/[M+H]^+^ against collision energy expressed in terms of the center-of-mass E_comδ_ (the maximum kinetic energy available for transfer into internal energy) were made. In the performed experiments, the collision energy is defined as:

$$E_{com\delta }={\delta E}_{lab}\frac{m_{g}}{m_{p}+m_{g}},$$

where E_lab_ stands for the laboratory collision energy (in this experiment it is equal to the cone voltage value), m_g_ stands for the mass of the neutral collision gas (for ‘in-source’ CID, it is N_2_ of M = 28 amu), m_p_ stands for the mass of the adduct and δ is the correction factor, which reflects the number of degrees of freedom (DOF = 3N − 6, N stands for the number of atoms in the analyzed ions) [40,41,42]. The adducts [**3**+dG+H]^+^ and [**3**+G+H]^+^ are the smallest of those analyzed, so these adducts were selected as references (DOF_[3+dG+H]+_ = 246, DOF_[3+G+H]+_ = 249). Therefore, for these adducts, δ = 1, and for the others, δ = DOF_ref_/DOF_adduct_. As the proton affinities of guanosine and deoxyguanosine are surely higher than those of phenolics, it can be taken for granted that the adducts are composed of neutral M molecules and protonated dG/G molecules and adducts decomposition consist in the formation of these species. In the applied cone voltage range (10, 15, 20, 25, and 30 V), the fragmentation of phenolic compounds was not observed, therefore the abundances of phenolic [M+H]^+^ ions increases. The analyzed adducts are formed due the weak non-covalent interactions, so for these adducts, in the applied cone voltage range, two opposite effects occur. Therefore, the comparison of the ions ratio [M+dG/G+H]^+^/[M+H]^+^ reflects the relative stabilities of the [M+dG/G+H]^+^ adducts.

**Figure 2:**
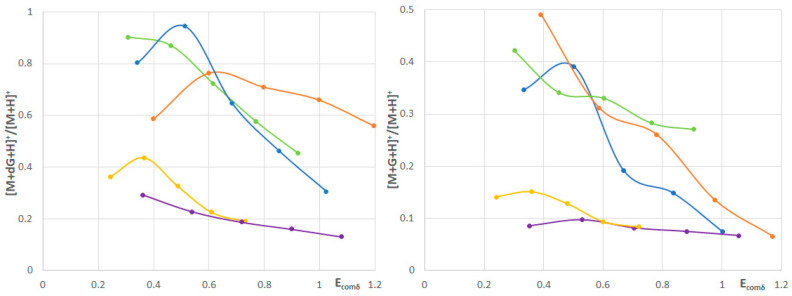
The plots of [M+dG+H]^+^/[M+H]^+^ and [M+G+H]^+^/[M+H]^+^ against E_comδ_. Blue—**1**, orange—**2**, green—**3**, violet—**4**, yellow—**5**.

The plots of [M+dG+H]^+^/[M+H]^+^ and [M+G+H]^+^/[M+H]^+^ against E_comδ_ are shown in Figure 2.

Notably, [**5**+dG/G+H]^+^ has a higher stability than [**4**+dG/G+H]^+^. It has already been established that the presence of sugar moiety increases the stability of the phenolic adducts with G-quadruplex/G-tetrad (sugar moiety is a source of hydrogen bonds) [43,44,45,46]. Therefore, the results obtained for standards **4** and **5** indicate that the approach used in this study works successfully.

According to the obtained results, compounds **1**–**3** form more stable adducts with deoxyguanosine and guanosine than standards **4** and **5** (Figure 2). It has already been found that 3-O-methylquercetin forms more stable adducts with G-tetrad than quercetin [47]; therefore, 3-*O*-methylquercetin glycoside (**1**) also forms more stable adducts with dG/G than quercetin glycosides (**4** and **5**). Although the decrease in the [M+dG/G+H]^+^/[M+H]^+^ ratio for **1**–**3** is higher than that for **4** and **5**, in the employed cone voltage range, the [M+dG/G+H]^+^/[M+H]^+^ ratio for **1**–**3** is always higher than that for **4** and **5** (Figure 2). The larger decrease in the [M+dG/G+H]^+^/[M+H]^+^ ratio for **1**–**3** is probably because some of the non-covalent interactions present in the adducts of **1**–**3** are weakened in the gas phase.

3-*O*-Methylquercetin is already known for its anticancer activities [25,26,28]. The high affinity of 3-*O*-methylquercetin-7-*O*-glucoside (**1**) toward deoxyguanosine and guanosine indicates that this 3-*O*-methylquercetin conjugate also has promising anticancer properties, namely as an effective G-quadruplex binding agent. Chlorogenic acid (3-caffeoylquinic acid) has shown notable binding properties toward G-quadruplex [23]. In this work, it is plausible that 3,5-di-O-caffeoylquinic acid (3) has stronger binding properties toward G-quadruplex, due to the presence of the second caffeoyl moiety (which can be an additional source of hydrogen bonds and π-stacking interaction). Kawain and its conjugates have shown a number of pharmacological properties (including anticancer) [48]; however, to the best of our knowledge, their binding property toward G-quadruplex has not been reported yet. A high affinity of 4′-hydroxydehydrokawain-4′-*O*-glucoside (**2**) proved in this study indicates that this class of compounds has this property and requires further study.

We are aware that high ligand affinity toward dG/G does not always imply high affinity toward G-quadruplex. Namely, the sites on dG/G molecules that can interact with the ligands may not interact with the ligand when the dG/G molecules form a quadruplex (in the G-quadruplex structure, a number of H/N/O atoms of dG/G are involved in the formation of hydrogen bonds). On the other hand, in the analyzed adducts, there are many kinds of interactions, which can also be expected for G-quadruplex (e.g., N3 involving interactions, hydrogen bonds with sugar moieties, pi-pi interactions), as shown by the performed molecular docking.

### 2.3. Molecular Docking

**Figure 3:**
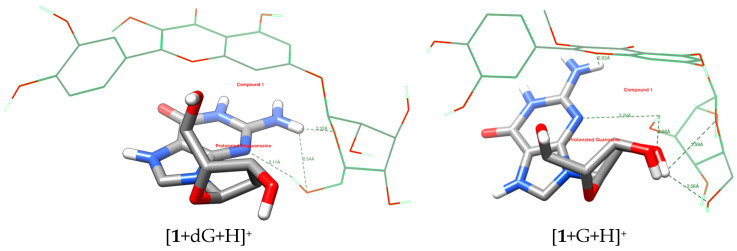
Potential hydrogen bond (green dashed lines) formation of **1** with protonated deoxyguanosine and guanosine.

**Figure 4:**
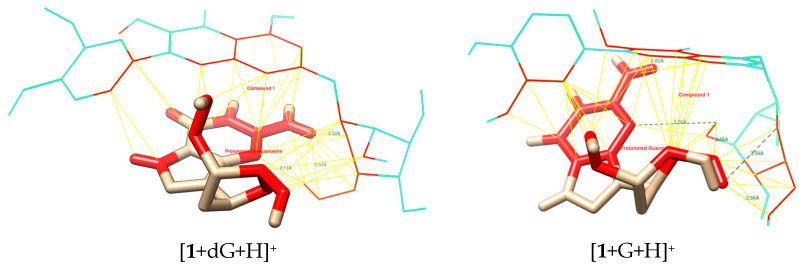
Potential interactions (especially pi-pi, yellow lines) of **1** with protonated deoxyguanosine and guanosine.

To gain some insight into the structures of the analyzed adducts, molecular docking was performed for the adducts. Of course, the structures obtained by molecular docking are the most stable (minimum energies), so they may not fully reflect those existing in the experimental conditions, which may exist at higher energy states. The obtained results are presented in Table 2 and Table 3, and Figure 3, Figure 4, Figures S5 and S6.

Table 2 includes binding energies of protonated guanosine and protonated deoxyguanosine, revealing that rutin (**5**) exhibits the highest binding energy to both protonated guanosine and deoxyguanosine (−3.5 kcal/mol). 3-*O*-Methylquercetin-7-*O*-glucoside (**1**) also shows favorable binding energies, with −3.3 kcal/mol and −3.2 kcal/mol, respectively. Compounds **2** and **4** display moderate interactions, ranging from −2.8 to −3.1 kcal/mol for both targets. Compound **3** displays the weakest interactions, with binding energies of −2.2 kcal/mol and −2.3 kcal/mol. Since **3** is the only one that does not contain sugar moiety, the obtained results indicate that this moiety is of key importance to obtain the low minimum energy of the adducts.

Table 3 outlines the number of potential hydrogen bonds between ligands and nucleosides, and Figure 3 and Figure S5 show the graphical visualization of the bonds. Compound **5** exhibits the highest H-bonding potential (six bonds with both targets). Compound **3** shows differential H-bonding (five with guanosine, three with deoxyguanosine). Compound **4** maintains consistent H-bonding (four bonds), while compounds **1** and **2** show the lowest H-bonding potential (three and two to three bonds, respectively).

Compound **5** shows strong binding energy (−3.5 kcal/mol) correlated with high H-bonding. Compound **1** exhibits strong binding despite having fewer H-bonds, suggesting dominant pi-pi interactions. Compound **3** has moderate-to-high H-bonds but weak binding, implying suboptimal geometry or steric hindrance. Guanosine’s 2′-OH group influences H-bonding, but consistent binding energies suggest that pi-pi interactions compensate for H-bond variations (Figure 4 and Figure S6).

## 3. Materials and Methods

### 3.1. Sample Preparation

The *Achyrocline satureioides* flowers (herbal tea, dietary supplement) were obtained from Diochi (Prague, Czech Republic), country of origin: Paraguay. A portion of 2 g was extracted with 10 mL of pure methanol, the sample was shaken at 500 rpm for 30 min (Vortex 3, IKA-Werke GmbH, Staufen, Germany), sonicated, and filtered through syringe filters with a pore size of 0.45 μm (Macherey-Nagel GmbH, Düren, Germany). Prior to the HPLC/ESI-MS analysis, the sample was further diluted at 1:1 in pure methanol (stored at 5 °C).

The deoxyguanosine (dG), guanosine (G), isoquercitrin (quercetin-3-*O*-glucoside—**4**), and rutin (**5**) were obtained from Sigma-Aldrich (Poznań, Poland). For the post-column addition experiment, the deoxyguanosine and guanosine solutions (both at concentration 10 mg/mL) were prepared in ammonia water solution (0.5 mM, taking into account the dG solubility properties) and in methanol, respectively. It is clear that the use of different solvents for the preparation of dG and G solutions excluded a comparison of the results obtained for these nucleosides. The solutions of **4** and **5** were prepared in methanol at a concentration of 10^−4^ mol/dm^3^.

### 3.2. HPLC-MS Analysis and Post-Column Addition Experiments

The HPLC-MS analyses were performed using a Waters Arc HPLC pump, a Waters SQD mass spectrometer (a single quadrupole type instrument equipped with electrospray ion source, Z-spray, Milford, MA, USA). The software used was MassLynx V4.2 SCN1046 (Milford, MA, USA). Using an autosampler, the sample solutions were injected into a C18 Atlantis T3 column (3 µm, 100 mm × 3 mm i.d.). The injection volume was 10 µL. The solution was analyzed by using the linear gradient of CH_3_CN-H_2_O at a flow rate of 0.7 mL/min The gradient started from 3% CH_3_CN—95% H_2_O with 2% of a 10% solution of formic acid in water, reaching 95% CH_3_CN after 10 min, and the latter concentration was maintained for 5 min. The ESI mass spectra were recorded in the *m*/*z* range 100–1000, in positive and negative modes simultaneously (during the HPLC-MS analyses, the mass spectrometer was switched in the fast mode between the positive and negative ion modes). The ESI source potentials were as follows: capillary, 3 kV; lens, 0.5 V; extractor, 4 V; cone voltage, 50 or 100 V. The cone voltage has the most profound effect on the mass spectra obtained. A cone voltage that is too low may cause a decrease in sensitivity (fewer ions reach the high vacuum region, especially those of high *m*/*z* values), and vice versa—a higher cone voltage can cause an increase in sensitivity. On the other hand, an increase in this parameter also leads to the so-called ‘in-source’ CID, which is of crucial importance with respect to the compounds’ identification. The source temperature was 120 °C, and the desolvation temperature was 300 °C. Nitrogen was used as the nebulizing and desolvating gas at flow rates of 100 and 300 L h^−1^, respectively.

Post-column addition HPLC-MS analysis was performed in the positive ion mode by using the same conditions as above, with the exception of cone voltages, which were lower (10, 15, 20, 25, and 30 V) to avoid the fragmentation of phenolic glycosides. In the positive ion mode at a cone voltage higher than 30 V, the weak glycosidic bond would be cleft. Since the compounds of interest were eluted in the range of 5–6 min (including standards), the guanosine and deoxyguanosine solutions were infused in the range of 4.5–6.5 min at a flow rate of 15 µL/min. To avoid or minimize the changes in relative ion abundances, e.g., because of the fluctuations in pressure inside the mass spectrometer, the adducts of each nucleoside were analyzed on the same day (in such a short period of time, the variations of relative ion abundances did not exceed 5%).

### 3.3. Molecular Docking

The protonated guanosine and deoxyguanosine structures were built using the Avogadro program 1.2.0 [49,50]. Subsequently, the structures were converted into pdbqt format, which is suitable for the molecular docking procedure in the AutoDock Vina 1.1.2 [51] program with the application of OpenBabel software 3.1.1 [52,53]. The results of AutoDock Vina algorithm execution were visualized using Chimera 1.16 software [54]. For the docking process for both protonated guanosine and deoxyguanosine, the following search space was used: center (x, y, z) (0, 0, 0), size (x, y, z) (40, 40, 40).

## 4. Conclusions

The described deoxyguanosine/guanosine post-column addition experiment enabled the fast evaluation of the phenolic compounds’ affinity toward these nucleosides. As these nucleosides are key building blocks of G-quadruplexes, the presented approach may be regarded as a very useful screening method in the search for effective G-quadruplex binders. It has been found that the presence of sugar moiety, as well as the methylation of the C3-OH group, increases the stability of the analyzed adducts, analogous to previous findings for G-quadruplex/G-tetrad adducts, which means that the method is successful.

The performed molecular docking has shown that the binding energy of the analyzed compounds toward deoxyguanosine/guanosine is a result of H-bonding and pi-pi interactions, i.e., multiple interactions are of key importance for the adducts’ stability. Compound 5 highlights H-bond/affinity correlation, while compound 1 shows that pi-pi interactions can compensate for fewer H-bonds.

The results of both the post-column addition experiment and the molecular docking suggest that the phenolic of *Achyrocline satureioides*, namely 3-*O*-methylquercetin-7-*O*-glucoside (**1**), is a very promising and effective G-quadruplex binder. To the best of our knowledge, *Achyrocline satureioides* has not yet been considered as a source of effective G-quadruplex ligands.

## Figures and Tables

**Table 1:** The results of cone voltage induced fragmentation obtained for compounds **1**–**3**.

Compound	Detected Ions *m*/*z*
3-*O*-methylquercetin-7-*O*–glucoside (**1**)	477 ([M–H]^−^), 462 ([M–H–CH_3_]^−•^), 315 ([aglycone–H]^−^), 300 ([aglycone–H–CH_3_]^−•^), 299 ([aglycone–H–CH_4_]^−^), 271 ([aglycone–H–CH_4_–CO]^−^); 479 ([M+H]^+^), 317 ([aglycone+H]^+^), 302 ([aglycone+H–CH_3_]^+•^)
4′-hydroxydehydrokawain-4′-*O*-glucoside (**2**)	451 ([M+HCOO]^−^), 441 ([M+Cl]^−^), 243 ([aglycone–H]^−^); 407 ([M+H]^+^), 245 ([aglycone+H]^+^)
3,5-di-*O*-caffeoylquinic acid (**3**)	515 ([M–H]^−^), 353 ([M–H–(OC–CH=CH–C_6_H_4_O_2_)]^−^), 191 ([M–H–(OC–CH=CH–C_6_H_4_O_2_)_2_]^−^ or [quinic acid–H]^−^), 173 ([quinic acid–H–H_2_O]^−^), 179 ([caffeoyl acid–H]^−^), 135 ([CH=CH–C_6_H_5_O_2_]^−^) 517 ([M+H]^+^), 163 ([OC–CH=CH–C_6_H_5_O_2_]^+^)

**Table 2:** The binding energies of compounds **1**–**5** to protonated deoxyguanosine and guanosine.

Compound	Binding Energy to Protonated Deoxyguanosine [kcal/mol]	Binding Energy to Protonated Guanosine [kcal/mol]
**1**	−3.2	−3.3
**2**	−2.8	−2.9
**3**	−2.3	−2.2
**4**	−3.0	−3.1
**5**	−3.5	−3.5

**Table 3:** The number of potential hydrogen bonds in the analyzed adducts.

Adduct	dG	G
[**1**+dG/G+H]^+^	2	3
[**2**+dG/G+H]^+^	3	3
[**3**+dG/G+H]^+^	3	5
[**4**+dG/G+H]^+^	4	4
[**5**+dG/G+H]^+^	6	6

## Data Availability

The raw data supporting the conclusions of this article will be made available by the corresponding authors on request.

## Supplementary Materials

The following supporting information can be downloaded at: https://www.mdpi.com/article/10.3390/ijms26094312/s1.
