# Supplementary material for: Post–Column Guanosine Addition as a Screening Tool in the Search for Effective G–Quadruplex Binders—A Case Study of Achyrocline satureioides Phenolic Compounds
Source: Int J Mol Sci. 2025 May 1;26(9):4312. doi: 10.3390/ijms26094312 (PMC12072449; doi:10.3390/ijms26094312)
Supplement: Supplementary file 1 [file ijms-26-04312-s001.zip › ijms-3591795-supplementary.pdf]

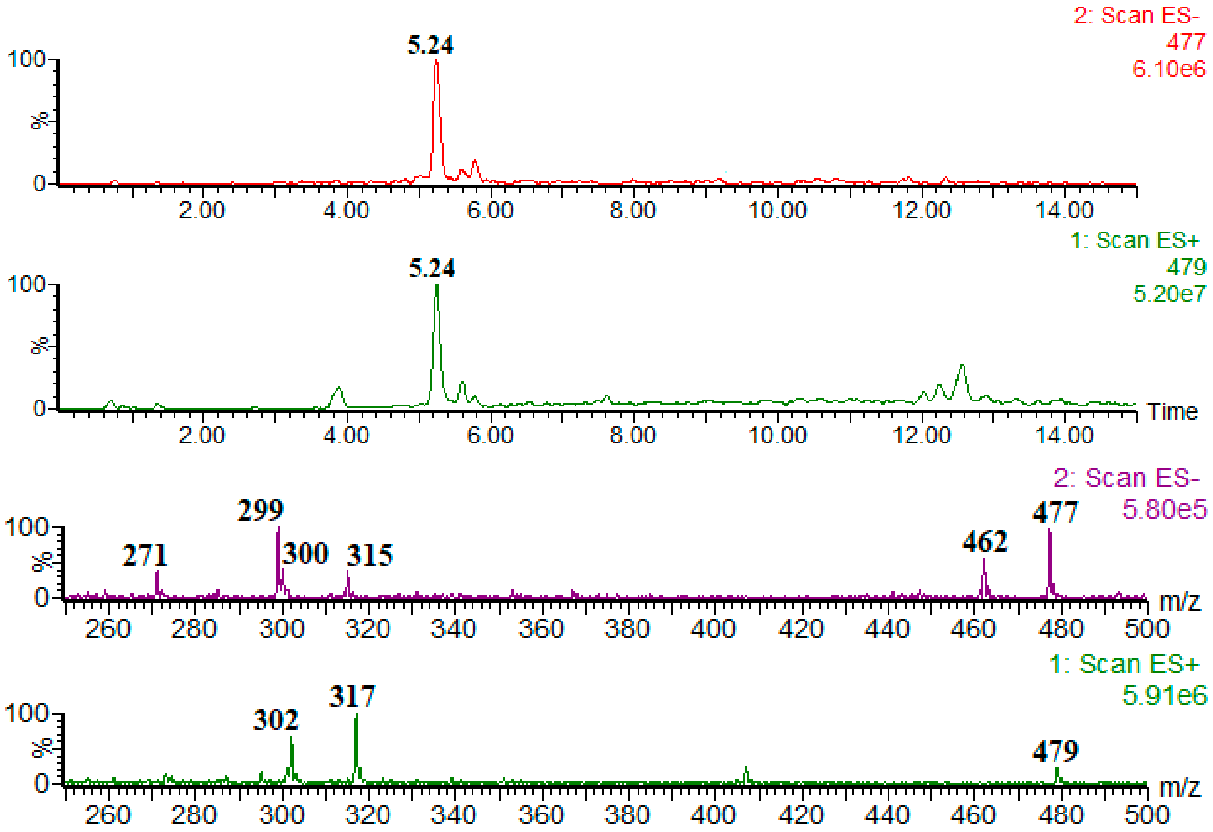

Figure S1. HPLC-MS data obtained for 3-*O*-methylquercetin-7-*O*–glucoside (**1**).

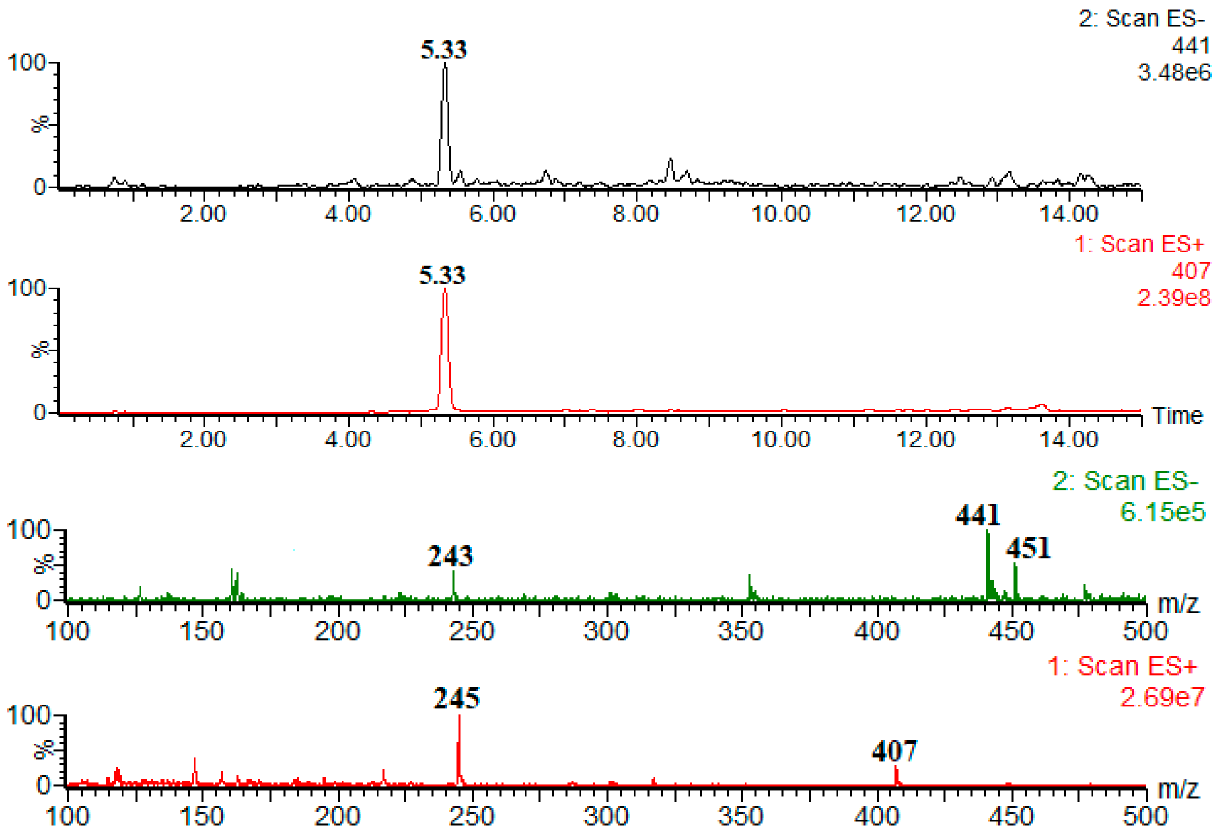

Figure S2. HPLC-MS data obtained for 4'-hydroxydehydrokawain-4'-*O*-glucoside (**2**).

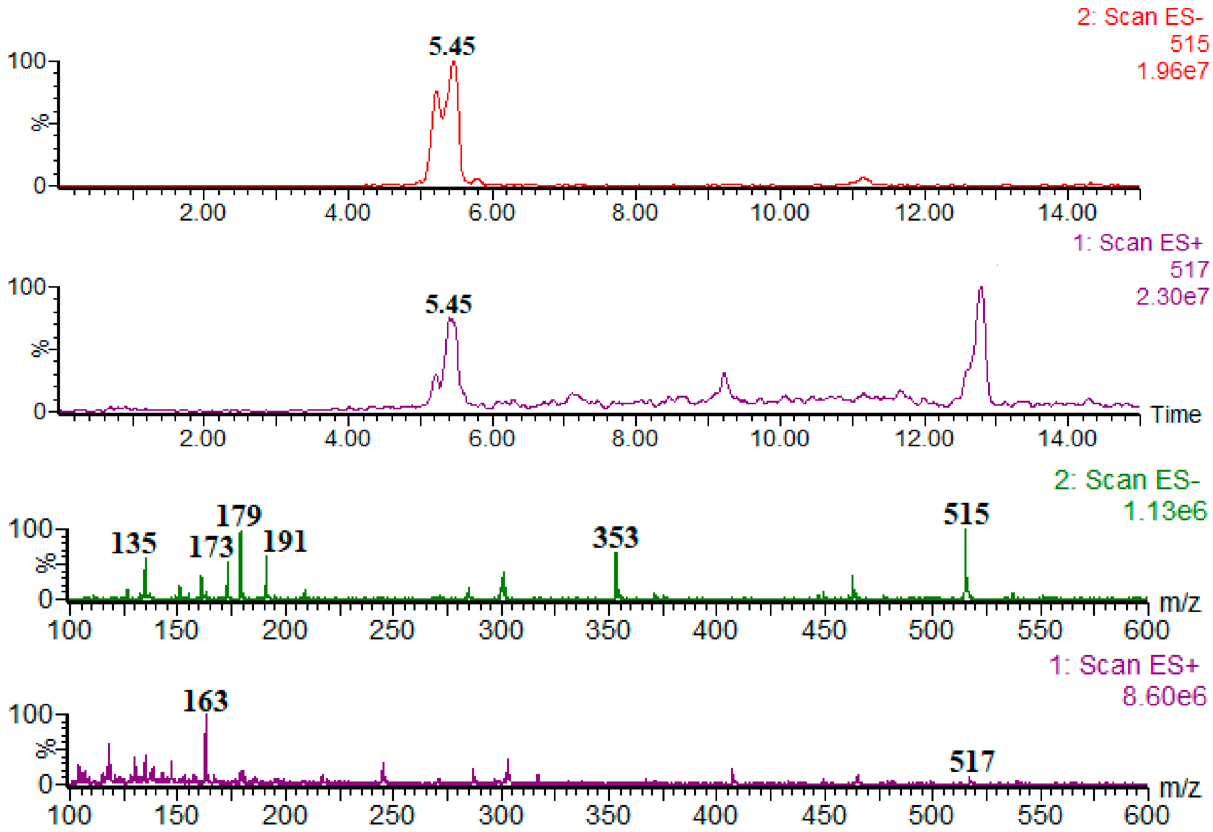

Figure S3. HPLC-MS data obtained for 3,5-di-*O*-caffeoylquinic acid (**3**).

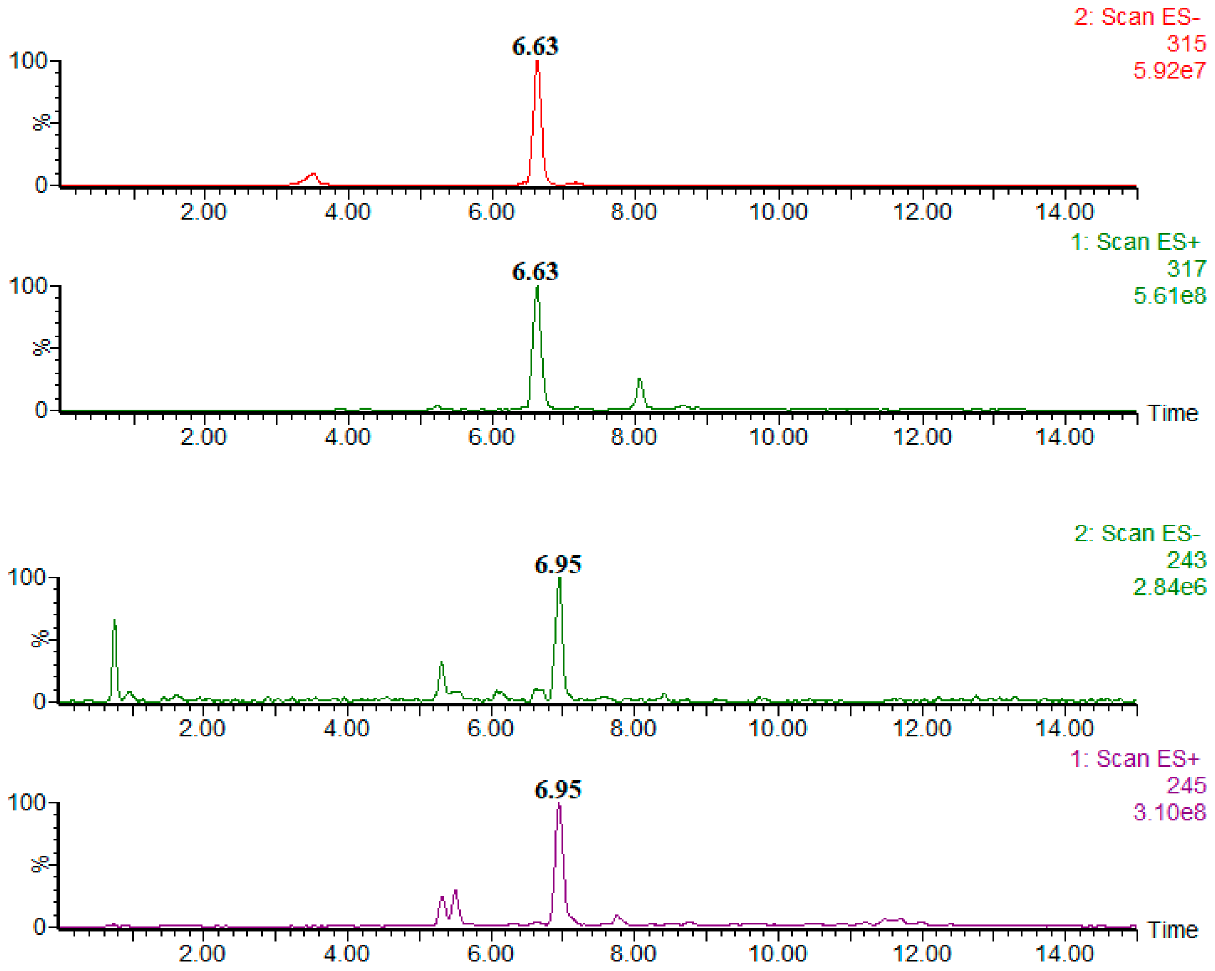

Figure S4. Single ion chromatograms of free aglycones of **1** and **2**, 3-*O*-methylquercetin at *m/z* 315 and 317, 4'-hydroxydehydrokawain at *m/z* 243 and 245.

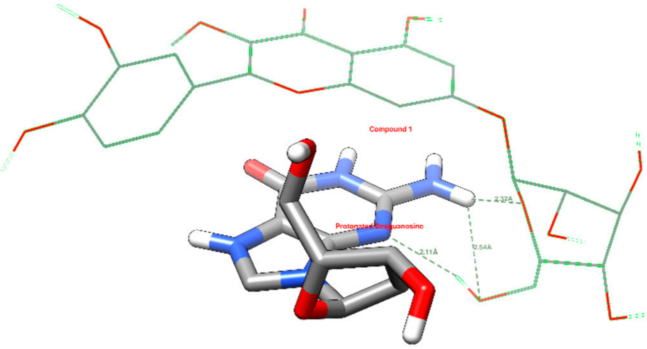

[**1**+dG+H]$^+$

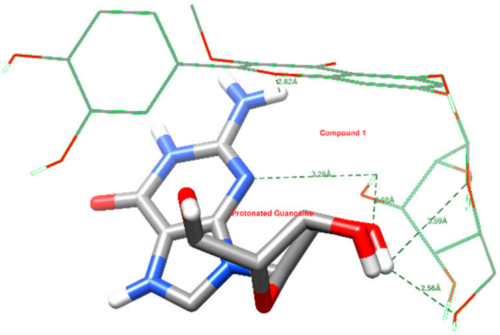

[**1**+G+H]$^+$

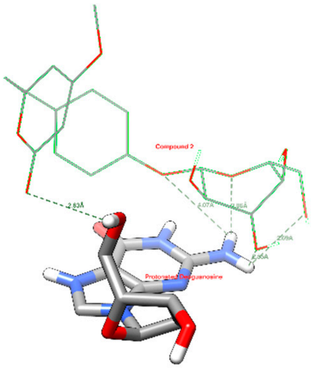

[**2**+dG+H]$^+$

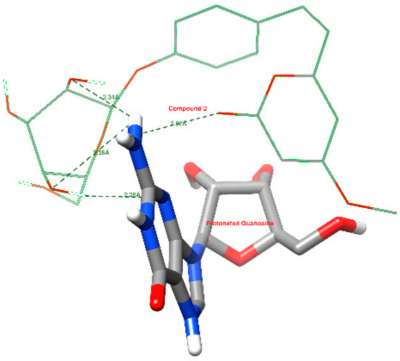

[**2**+G+H]$^+$

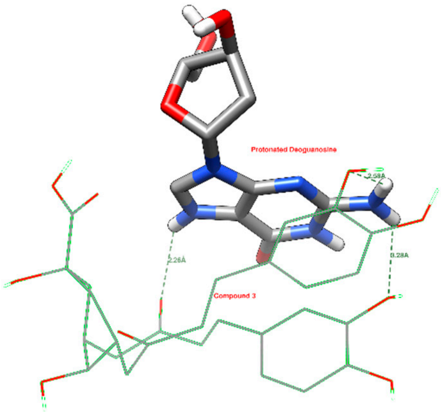

[**3**+dG+H]$^+$

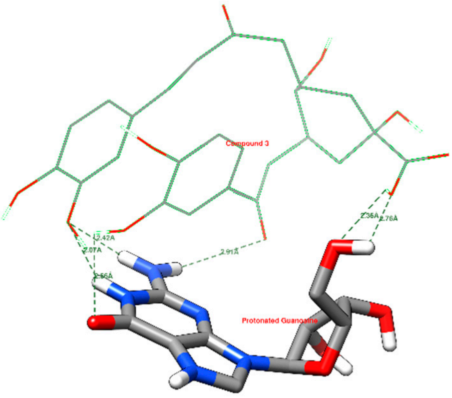

[**3**+G+H]$^+$

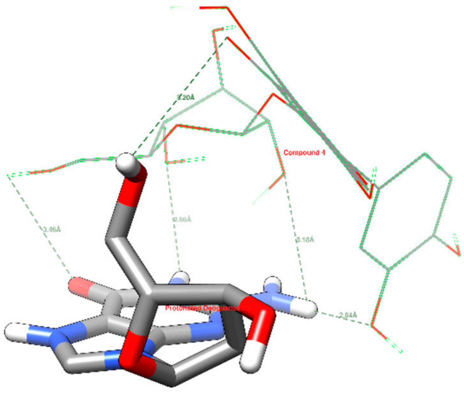

[**4**+dG+H]$^+$

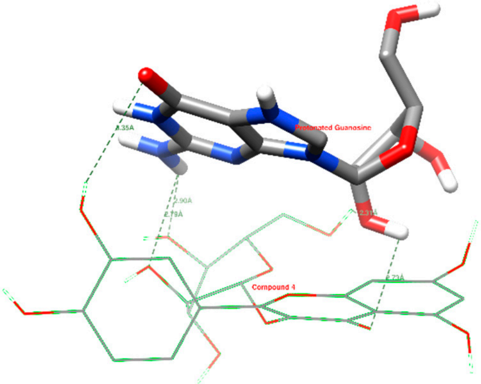

[**4**+G+H]$^+$

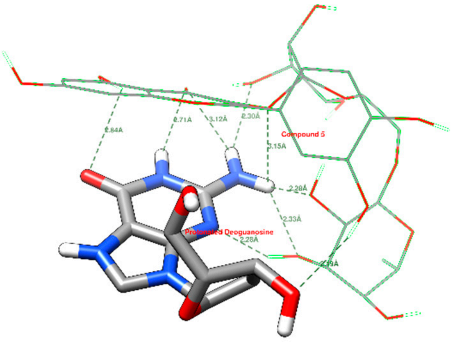

[**5**+dG+H]$^+$

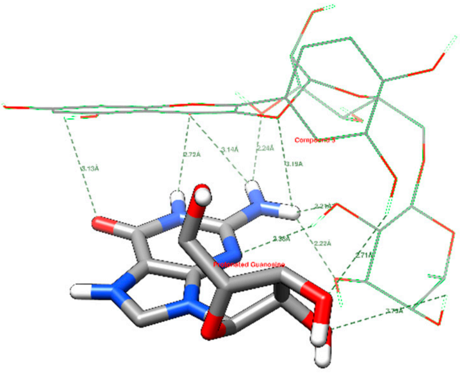

[**5**+G+H]$^+$

Figure S5. Potential hydrogen bonds formation (green dashed lines) of **1-5** with protonated deoxyguanosine and guanosine.

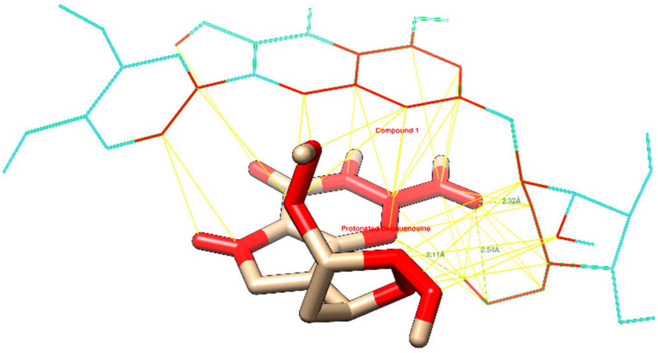

[**1**+dG+H]$^+$

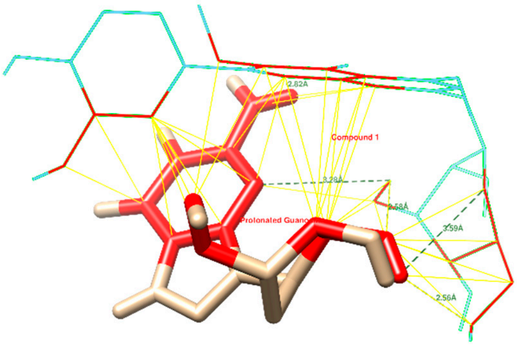

[**1**+G+H]$^+$

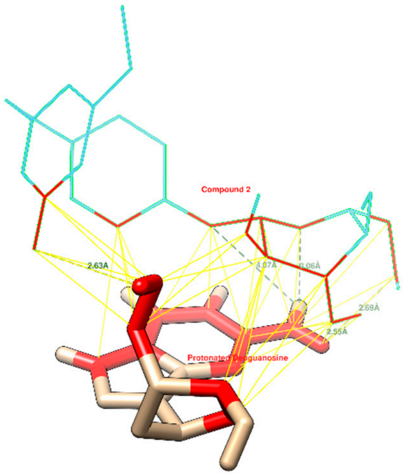

[**2**+dG+H]$^{+}$

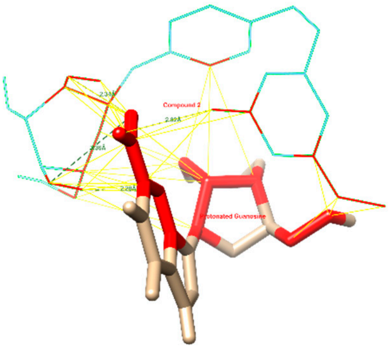

[**2**+G+H]$^{+}$

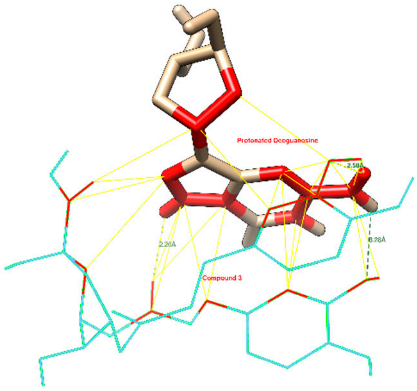

[**3**+dG+H]$^{+}$

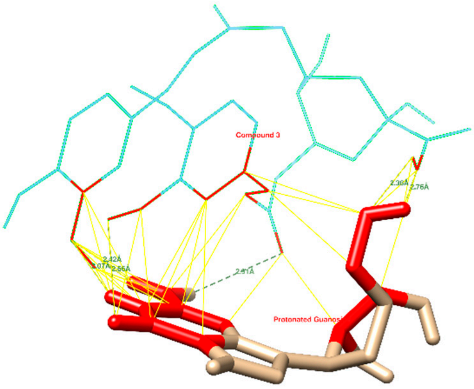

[**3**+G+H]$^{+}$

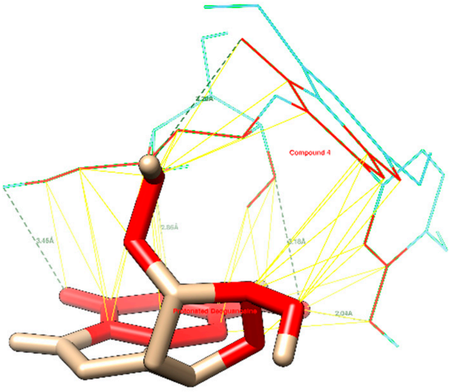

[**4**+dG+H]$^{+}$

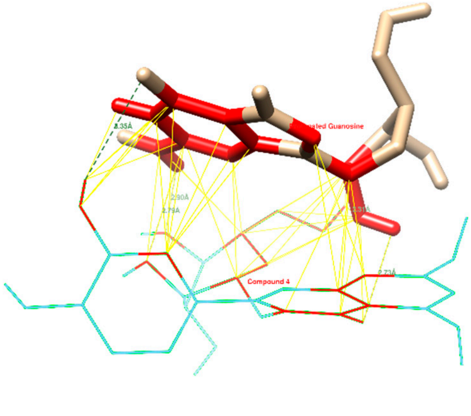

[**4**+G+H]$^{+}$

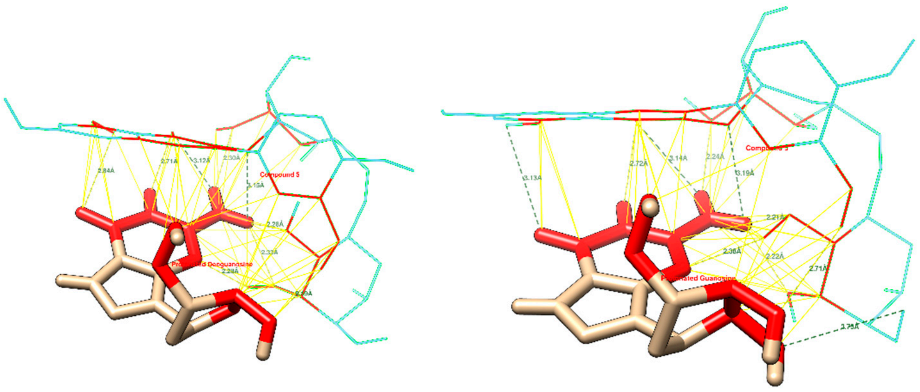

$[\mathbf{5}+dG+H]^+$ $[\mathbf{5}+G+H]^+$

Figure S6. Potential interactions (especially pi-pi, yellow lines) of **1**-**5** with protonated deoxyguanosine and guanosine.
